# Indirect Effects of Wnt3a/β-Catenin Signalling Support Mouse Spermatogonial Stem Cells In Vitro

**DOI:** 10.1371/journal.pone.0040002

**Published:** 2012-06-28

**Authors:** Jonathan R. Yeh, Xiangfan Zhang, Makoto C. Nagano

**Affiliations:** Division of Experimental Medicine, Department of Obstetrics and Gynecology, McGill University, Montreal, Quebec, Canada; National Cancer Institute, United States of America

## Abstract

Proper regulation of spermatogonial stem cells (SSCs) is crucial for sustaining steady-state spermatogenesis. Previous work has identified several paracrine factors involved in this regulation, in particular, glial cell line-derived neurotrophic factor and fibroblast growth factor 2, which promote long-term SSC self-renewal. Using a SSC culture system, we have recently reported that Wnt5a promotes SSC self-renewal through a β-catenin-independent Wnt mechanism whereas the β-catenin-dependent Wnt pathway is not active in SSCs. In contrast, another study has reported that Wnt3a promotes SSC self-renewal through the β-catenin-dependent pathway, as it can stimulate the proliferation of a spermatogonia cell line. To reconcile these two contradictory reports, we assessed Wnt3a effects on SSCs and progenitor cells, rather than a cell line, in vitro. We observed that Wnt3a induced β-catenin-dependent signalling in a large subset of germ cells and increased SSC numbers. However, further investigation revealed that cell populations with greater β-catenin-signalling activity contained fewer SSCs. The increased maintenance of SSCs by Wnt3a coincided with more active cell cycling and the formation of germ cell aggregates, or communities, under feeder-free conditions. Therefore, the results of this study suggest that Wnt3a selectively stimulates proliferation of progenitors that are committed to differentiation or are in the process of exiting the SSC state, leading to enhanced formation of germ cell communities, which indirectly support SSCs and act as an in vitro niche.

## Introduction

Spermatogonial stem cells (SSCs) are the foundation of life-long spermatogenesis. SSCs have the unique ability to maintain the stem cell pool through self-renewing divisions as well as to generate daughter cells committed to differentiation thereby producing mature sperm. This fate decision is believed to be tightly regulated during steady-state spermatogenesis and occurs in a specialized microenvironment, referred to as the niche, which supports SSCs [1]. The concept of a niche is well demonstrated in the Drosophila testis where somatic cells, hub cells, are located at the distal tip of the testis and are responsible for maintaining the stemness of germ-line stem cells through direct contact [2]. Migration away from contact with hub cells leads to differentiation toward sperm. Interestingly, it has been shown that differentiated germ cells can regain stem cell activity upon homing back to hub cells suggesting the inductive nature of the Drosophila testis niche [3].

Such a defined niche has not been characterized in mammalian testes. Rather, SSCs reside along the basement membrane of the seminiferous tubule in close contact with Sertoli cells, the supporting somatic cells [1]. A great deal of work has been performed to identify how Sertoli cells support SSCs. To date, a handful of factors derived from testicular somatic cells, such as Sertoli cells, peritubular myoid cells, and Leydig cells, have been demonstrated to influence SSC renewal, in particular glial cell-line derived neurotrophic factor (GDNF), fibroblast growth factor 2 (FGF2), colony stimulating factor 1, and Wnt5a [4], [5], [6], [7]. In contrast, knowledge of factors associated with SSC commitment to differentiation is limited. In addition, SSCs are not only in close contact with Sertoli cells but also with other types of spermatogonia in the niche. Therefore, the possibility exists that non-stem spermatogonia can communicate with SSCs and contribute to the regulation of SSC activity. However, this possibility has not been investigated.

Although mechanisms that control SSC fate are not completely understood, SSCs can be expanded in vitro under well defined conditions. In the presence of GDNF and FGF2, SSCs proliferate in vitro and form distinct accumulations of SSCs and daughter spermatogonia, which we term “clusters” [4], [8], [9]. Interestingly, previous work has shown that SSCs are a minority population among cluster cells, and the reported percentages of SSCs in clusters are relatively constant (1–3%) across several studies [5], [8], [9]. These observations raise a possibility that clusters constitute a society of male germ cells in vitro in which cells communicate with one another, thereby regulating the proportion of SSCs in the community [10].

In a previous study, we reported the expression of various Wnts in mouse testes and feeder cells used in SSC cultures [7]. Wnts are a family of lipid-modified, secreted glycoproteins with diverse functions in embryogenesis, tumorigenesis, as well as stem cell proliferation and differentiation [11], [12]. In general, Wnt proteins can activate two classes of signalling cascades. The better characterized canonical (β-catenin) pathway involves Wnt stimulation with its receptor Frizzled and co-receptor low density lipoprotein-related protein (LRP) 5/6, ultimately resulting in interaction between β-catenin and members of the T-cell factor/Lymphoid enhancer factor (TCF/LEF) transcription factor family in the nucleus and modulation of target gene transcription. In contrast, non-canonical pathways involve a wide host of mediators that do not act through β-catenin.

Previous studies have demonstrated the importance of Wnt signalling in the niches of various adult stem cell types [13]. In vitro, β-catenin signalling promotes the expansion of phenotypically-defined hematopoietic stem cells (HSCs), neural stem cells (NSCs), and intestinal stem cells (ISCs) [14], [15], [16]. However, further studies show that HSCs can self-renew in the absence of β-catenin [17] and while constitutive β-catenin-signalling can expand HSCs in vitro, these cells do not retain the functional ability to differentiate into blood lineages [18]. Moreover, β-catenin signalling appears to be dispensable for NSCs and rather supports the adhesion and survival of progenitor cells in neurospheres [19]. Stem cells are defined functionally; therefore, these studies serve to highlight the importance of assessing pathway activation effects on stem cell activity in a functional manner.

Recently, we identified Wnt5a as a novel factor, expressed by feeder cells and Sertoli cells, that supports SSC renewal [7]. Using the functional transplantation assay for SSCs, we demonstrated that Wnt5a acts through a non-canonical mechanism and promotes SSC survival and self-renewal. Furthermore, the β-catenin pathway was not activated in SSCs and spermatogonia with active β-catenin signalling had committed to differentiation. In contrast, a study by Golestaneh et al. [20] has shown that Wnt3a stimulates the β-catenin pathway and leads to proliferation of an SV40-transformed spermatogonia cell line. However, the identity of this cell line as SSCs has not been examined functionally; i.e., the ability of these cells to regenerate and maintain spermatogenesis has not been demonstrated.

In an attempt to reconcile these two contrasting results and to better understand the role of Wnt/β-catenin-signalling in controlling SSCs and progenitor spermatogonia, we set out to functionally assess the effect of Wnt3a using SSC culture. We hypothesized that in accordance with our previous work Wnt3a would activate β-catenin signalling and reduce SSC numbers in vitro. However, Wnt3a surprisingly led to a significant increase in SSC numbers. We also observed that Wnt3a stimulated the β-catenin pathway in a large subset of cluster cells. These results appeared to contradict the findings of our previous study. However, further investigation demonstrated that although Wnt3a stimulated β-catenin signalling in a large population of germ cells, this population contains markedly reduced numbers of SSCs suggesting that the increase in SSC numbers occurred in cluster cells in which the β-catenin pathway was not activated. In addition, Wnt3a-induced increase in SSC numbers correlated with the increased formation of cell aggregations and cell-cell associations. Therefore, our study suggests that Wnt3a may regulate SSC activity indirectly by acting on committed daughter cells in vitro.

## Results

### Wnt3a Increases SSC Numbers in vitro

**Figure 1:**
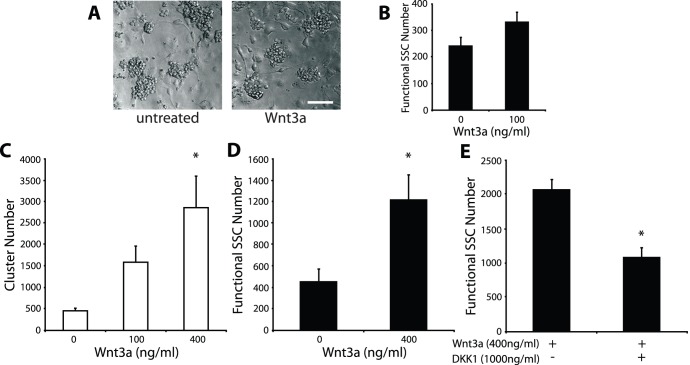
Wnt3a increases SSC numbers under feeder-free conditions. (**A**) Germ cell clusters (left) and after 6 day treatment with Wnt3a (right) (**B**) Quantification of SSCs following Wnt3a treatment measured by spermatogonial transplantation. Results of the cluster-forming (**C**) and transplantation (**D**) assays for SSC quantification, after feeder-free culture with Wnt3a. A significant increase in SSC numbers is detected with Wnt3a treatment by both assays. (**E**) Spermatogonial transplantation results following the addition of the β-catenin signalling inhibitor, DKK1, to Wnt3a-treated cultures. Inhibition of the β-catenin pathway inhibits the effect of Wnt3a on SSC activity. Scale bar: 75 µm.

A recent study has described the ability of Wnt3a to activate β-catenin signalling and stimulate proliferation in an SV40-transformed spermatogonia cell line [20]. This cell line is immunophenotypically similar to SSCs but has not been functionally tested as SSCs using spermatogonial transplantation. Therefore, whether Wnt3a affects SSCs in a similar manner to this cell line is unknown. To address this, we initially used an in vitro culture system in which SSCs can be expanded. This culture system uses the growth factors GDNF and FGF2 and a layer of mitotically inactivated mouse embryonic fibroblasts (STO cells) as feeder cells [4], [8]. Under these conditions, SSCs and their daughters can be expanded over a long period as distinct communities, termed “clusters” (Fig. 1A), of which SSCs comprise the minority population [5], [9]. Recombinant Wnt3a was added to B6ROSA clusters for one week and clusters developed similarly under both conditions (Fig. 1A). We transplanted treated clusters into the testes of infertile recipient mice to assess Wnt3a effects on SSC activity. Using this spermatogonial transplantation technique, SSCs normally hidden among the cells of a cluster can be unequivocally detected by their ability to regenerate spermatogenesis. Two months following transplantation, recipient mouse testes were analyzed to count colonies of regenerated spermatogenesis. We observed that Wnt3a led to a 1.4-fold increase in numbers of functional SSCs, but this increase was deemed to be not significantly different (Fig. 1B), leaving the result of Wnt3a on SSC activity somewhat ambiguous.

Previously, we reported the expression of other Wnt molecules by our STO feeder cells. To circumvent potential indirect effects via feeder cells, cluster cells were removed from feeder cells through gentle pipetting and cultured under feeder-less conditions on Matrigel-coated plates for a short duration (4 days) with growth factors and in the presence or absence of Wnt3a. Since our STO feeder cells are critical for cluster formation and SSC proliferation, clusters do not form under these conditions. Following treatment, cluster cells were recovered from feeder-less conditions and cultured back onto feeder cells to induce cluster formation. The number of clusters that form in this cluster-formation assay correlates to functional SSC numbers, thereby providing a short-term method to quantify SSCs [8]. Interestingly, we observed a dramatic dose-dependent increase in cluster-formation ability with the addition of Wnt3a (Fig. 1C). To verify this Wnt3a effect on functional SSCs, treated cluster cells were subjected to spermatogonial transplantation. We confirmed that the addition of Wnt3a led to a significant 2.7-fold increase in colony numbers (Fig. 1D), demonstrating that Wnt3a promotes SSC activity in vitro. Finally, we added the β-catenin signalling-specific inhibitor Dickkopf1 (DKK1) to Wnt3a-treated cultures and observed a significant decrease in colony numbers (Fig. 1E). Thus, these results indicate that the effects of Wnt3a on SSC activity are mediated by the β-catenin pathway.

### Wnt3a Activates β-catenin Signalling in a Subset of Cluster Cells and Reduces SSC Activity in these Cells

**Figure 2:**
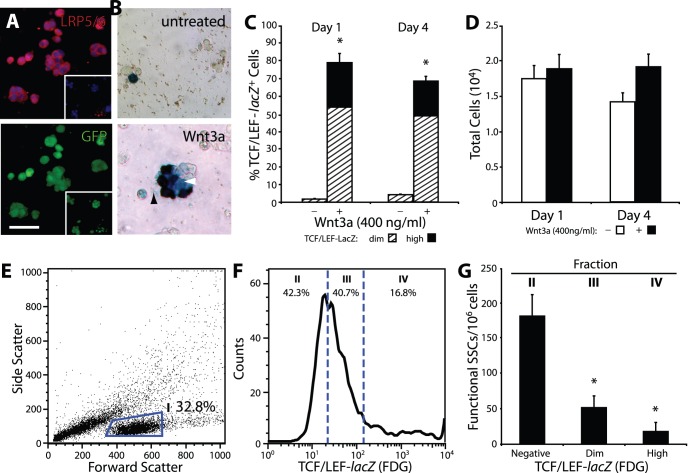
β-Catenin signalling cells increase with Wnt3a but have reduced SSC activity. (**A**) Immunofluorescent staining for LRP5/6 (top) and corresponding image showing B6GFP-derived cluster cells (bottom) indicate all cluster cells express LRP5/6. (Inset) Representative negative staining control. (**B**) TCF/LEF-*lacZ* cluster cells after 4 days under feeder-free conditions (top) and after Wnt3a treatment (bottom). Three populations emerge: β-Catenin signalling negative, dim (as indicated by white arrowhead), and high (as indicated by black arrowhead). (**C**) Wnt3a increases β-catenin signalling positive cells, however, proportions are maintained throughout the course of culture. (**D**) Total cell numbers do not change significantly upon Wnt3a stimulation. In the absence of Wnt3a, cell numbers tend to decrease slightly. (**E**) Flow cytometric scatter-plot assessing cell morphology. Cluster cells are found in Fraction I. (**F**) A representative FACS histogram sub-dividing Fraction I into β-catenin signalling negative (II), dim (III), and high (IV) fractions. (**G**) Concentration of SSCs in each fraction, measured by spermatogonial transplantation. A decrease in SSC activity is observed with an increase in β-catenin signalling intensity. Asterisks indicate significance from fraction II. Scale bar: (A–B) 50 µm, inset: 100 µm.

Previously, we demonstrated that Wnt5a supported SSCs through a non-canonical mechanism, whereas Wnt3a has been reported to act primarily through β-catenin signalling [7]. Immunoflourescent staining for the Wnt co-receptor LRP5/6 showed that all cluster cells express this protein (Fig. 2A). LRP5/6, in conjunction with the Wnt receptor, Frizzled, is necessary to transduce the Wnt/β-catenin signal. Frizzled protein expression has been reported on cluster cells [7]; therefore, all cluster cells appear able to signal through the β-catenin pathway. To confirm if Wnt3a indeed activates the β-catenin signalling pathway in cluster cells, we used established cluster cells derived from transgenic reporter mice (TCF/LEF-*lacZ* mice). These mice carry the *lacZ* reporter gene downstream of TCF/LEF binding sites, allowing faithful monitoring of β-catenin signalling activation [21], [22]. TCF/LEF-*lacZ* cluster cells were treated with Wnt3a on Matrigel, and the presence of β-catenin signalling cells were visually quantified after 4 days. On day 1, we detected a dramatic increase in the percentage of β-catenin signalling cells compared to untreated control cluster cells (Wnt3a vs. untreated; 79.0±3.6 vs. 1.8±0.2%) (Fig. 2B, C). Interestingly, the levels of β-catenin signalling activation varied in these cells with a gradient from moderate to robust lacZ expression (Fig. 2B, C). By day 4 of culture, we noted that this proportion of TCF/LEF-*lacZ* dim and high cells remained constant. We did not detect a significant change in total cell numbers upon Wnt3a stimulation under this feeder-free condition (Fig. 2D). β-Catenin signalling cells remained the majority population on day 4 and were significantly greater in number in Wnt3a-treated culture compared to untreated control culture (Wnt3a vs. untreated; 68.3±2.4 vs. 3.8±0.5%). These results indicate that Wnt3a activates the β-catenin pathway in cluster cells.

Previously, we demonstrated that global β-catenin pathway activation in all cluster cells using lithium chloride led to a loss in SSC activity suggesting that pathway activation may be associated with differentiation [7]. We therefore asked whether cluster cells stimulated by Wnt3a (i.e. an extracellular Wnt ligand vs. a global activator such as lithium) similarly lost SSC activity. To this end, TCF/LEF-*lacZ* cluster cells were cultured feeder-free in the presence of Wnt3a. Four days later, these treated cluster cells were recovered and reacted with a fluorescent vital β-galactosidase substrate (FDG) and then separated via FACS according to staining levels (Fig. 2E, F). Flow cytometric histograms of FDG activity were gated into three fractions, TCF/LEF-*lacZ* signalling negative (42.8±4.6%, Fraction II, Fig. 2F), dim (38.4±1.7%, Fraction III), and high expressing (18.7±3.4%, Fraction IV). The three fractions were isolated and separately transplanted into recipient testes to assess the SSC activity of each population. We observed that TCF/LEF-*lacZ* negative cells were highly enriched in functional SSCs (181.5±30.4 functional SSCs per 10^6^ cells), while β-catenin signalling cells had minimal SSC activity, similar to our previous observation (Fig. 2G) [7]. The TCF/LEF-*lacZ* ^dim^ population was composed of ∼50 functional SSCs per 10^6^ cells, while TCF/LEF-*lacZ* ^high^ had ∼18 functional SSCs per 10^6^ cells showing a trend of declining SSC frequency with an increase in β-catenin signalling intensity. Additionally, we performed quantitative PCR to examine the expression levels of markers for SSCs and differentiating spermatogonia (Fig. S1). We observed a trend showing decreased expression of Plzf in TCF/LEF-*lacZ* ^high^ cells and increased expression of Ngn3 and c-Kit in TCF/LEF-*lacZ* ^dim/high^, however no significance was detected. Therefore, these results collectively demonstrate that Wnt3a stimulates the β-catenin pathway in a subset of cluster cells but the population of functional SSCs is significantly diminished as β-catenin signalling is activated.

### Wnt3a Stimulates Active Cycling of β-catenin Signalling Cells and Leads to Cluster-like Aggregations under Feeder-free Conditions

**Figure 3:**
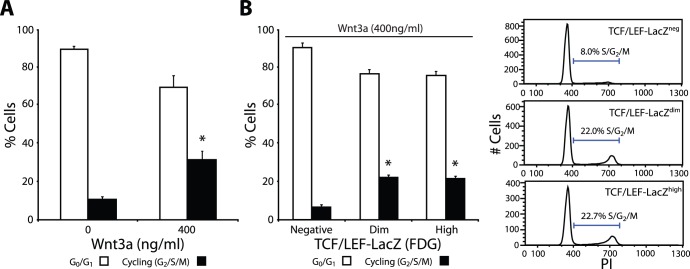
Wnt3a stimulates cluster cell cycling. (**A**) Percentage of non-cycling (G0/G1; open bars) and actively cycling (S/G2/M; filled bars) cells following Wnt3a stimulation. (**B**) Percentage of cycling cells in β-catenin signalling populations following Wnt3a stimulation. Representative flow cytometric histograms show β-catenin signalling positive cells are actively cycling after Wnt3a stimulation.

Since a previous study reported that proliferation of a spermatogonia cell line was stimulated by Wnt3a [20], we analyzed cell cycle profiles of cluster cells after Wnt3a treatment using propidium iodide staining and flow cytometry. Cluster cells were treated with or without Wnt3a overnight, under feeder-free conditions, and profiles were assessed the next day. We observed that most cluster cells are not actively cycling (G0/G1 phase) (Fig. 3A). However, following Wnt3a treatment we observed a significant 3-fold increase in the percentage of actively cycling cells (in S, G2, & M phase). We next asked whether active cycling is induced equally in both β-catenin signalling and non-signalling cells. To this end, TCF/LEF-*lacZ* clusters were stimulated with Wnt3a and separated via flow cytometry. Cell cycle profile analysis of each fraction showed that the fraction of actively cycling cells was markedly higher in β-catenin signalling cells than in non-signalling cells (negative: 6.7±1.3%, dim: 21.9±1.9%, high: 21.8±1.4%), (Fig. 3B). These results indicate that Wnt3a activates the β-catenin pathway in a select population of cluster cells and drives their cell cycle.

**Figure 4:**
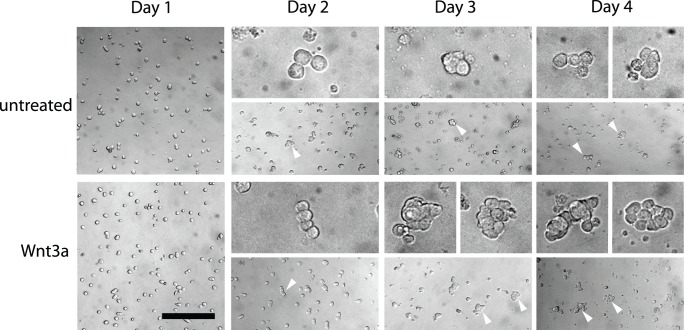
Wnt3a leads to increased “cluster-like” aggregations. Representative brightfield photomicrographs of cluster cells following control or Wnt3a treatment at each day of culture. At day 1 after seeding, cluster cells are arranged predominantly as singles or doublets under both conditions. At day 2, most cells remain as singles and doublets in both conditions but longer chains are more easily observable (arrowheads; upper panels are higher magnification). In day 3 control cultures, most cells remain as singles but small clumps are occasionally observed (arrowhead). In Wnt3a-treated cultures, larger clumps are observable by day 3 (arrowheads). On day 4, large “cluster-like” cell aggregations are found throughout Wnt3a-treated cultures (arrowheads), while control cultures are mostly singles and infrequently show small cell accumulations or the occasional cell chain (arrowheads) but lack large cell accumulations. Upper panels show higher magnification images. Scale bar: 50 µm, upper panels: 12.5 µm.

**Figure 5:**
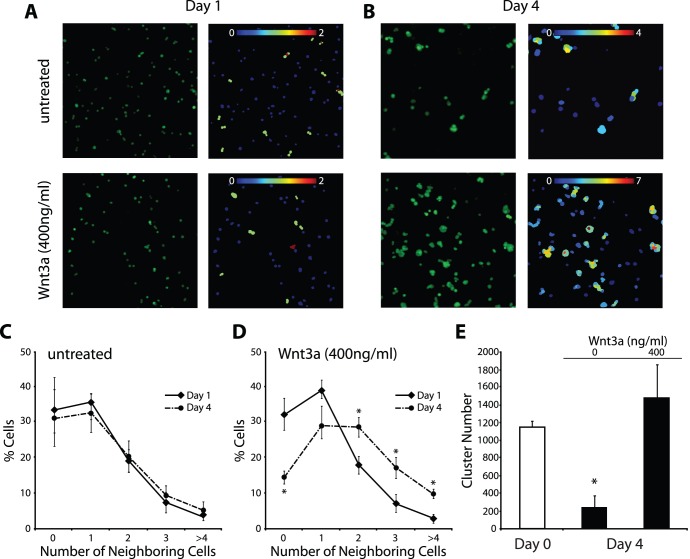
Wnt3a leads to increased cell-cell interactions. Representative fluorescent photomicrographs (left) and corresponding CellProfiler Image analysis (right) of B6GFP cluster cells following control or Wnt3a treatment after 1 day (**A**) and 4 days (**B**). The color gradient labels individual cells according to the number of directly-contacted neighboring cells. (**C, D**) Percentage of clustering cells after 1 day (solid line) and 4 days (dashed line) following control (C) and Wnt3a treatment (D). Wnt3a stimulates cells to cluster, while most cells remain as singles or doublets in controls. (**E**) Cluster-formation assay results showing SSC numbers over the course of feeder-free culture with or without Wnt3a. Increased SSC maintenance is observed with Wnt3a.

Over the course of our short-term feeder-free culture, we observed that “cluster-like” accumulations formed in the presence of Wnt3a, while in its absence most cells remained as singles or doublets (Fig. 4). Therefore, to quantify whether Wnt3a led to an increase in the frequency of directly-contacted, aggregating cells, we employed Cell Profiler image analysis software (Broad Institute) [26] and monitored the daily growth of B6GFP cluster cells in the presence of Wnt3a, comparing them to untreated controls. We utilized an algorithm that identified individual cells based on GFP expression and the presence of a DNA dye. This algorithm then measured how many adjacent, neighboring cells each cell directly contacts, thereby identifying the frequency of cell aggregation. The results showed that in the absence of Wnt3a, most cells remain with 0–1 connected cells (i.e. singles or doublets) throughout the course of culture (Fig. 5A, B, higher magnification image is found in Fig. S2). In contrast, Wnt3a-treated cells started as singles or doublets at day 1 of culture, similar to controls. However, the frequency of singles/doublets declined throughout the course of culture until most cells had at least 1–2 neighboring cells, while only ∼14% of cells are classified as singles by day 4 (Fig. 5C, D). Furthermore, Wnt3a-treated cultures showed 10% of cells with 4 or more neighboring cells by day 4, which represents a significant increase compared to untreated controls where only 5% of cells have 4 or more neighbors. Therefore, these results show that Wnt3a stimulation leads to the formation of cluster-like cell aggregations under feeder-free conditions.

Finally, we attempted to determine if Wnt3a leads to SSC expansion over the course of 4 day culture, under feeder-free conditions. Using the cluster-formation assay, we compared the numbers of SSCs at the beginning (day 0) to the end (day 4) of feeder-free culture with or without Wnt3a. From this comparison we can determine if SSC numbers increase, decrease, or are simply maintained. From day 0 to day 4, SSC numbers increased 1.3-fold with Wnt3a, which was deemed not significant (Fig. 5E). In the absence of Wnt3a cluster-forming cells decreased dramatically. Therefore, these results suggest that development of cell-cell associations supports the maintenance of SSC activity over the course of feeder-free culture.

## Discussion

The results of this study indicate that Wnt3a can activate the β-catenin pathway in a subset of cluster cells but the majority of these cells are not SSCs, which is consistent with our previous study [7]. Interestingly, Wnt3a treatment leads to an overall increase in SSC numbers. We observe that β-catenin pathway activation leads to increased cell cycle activity and more robust cell-cell association upon Wnt3a treatment, resulting in an increase in SSC maintenance. Thus, these results collectively suggest that Wnt3a increases SSC numbers indirectly through stimulating the formation of the cluster community, raising the possibility that the presence of committed cells might be important to maintain SSCs.

During steady-state spermatogenesis, SSCs are believed to represent a population of the most primitive A_single_ spermatogonia. However, following injury or depletion it has been shown that more differentiated A_paired_ or A_aligned_ spermatogonia may revert to an SSC fate [27], [28]. Accordingly, cluster cells expressing the differentiation marker c-kit are observed to retain SSC regenerative ability equal to their c-kit counterparts [29], implying that all cells within the cluster community may retain a level of SSC activity. We observed that the majority of functional SSCs were TCF/LEF-*lacZ* non-signalling cells and that SSC activity is lost as activation levels of β-catenin signalling increased (Fig. 2). Therefore, it is possible that cluster cells may be induced for gradual commitment to differentiation upon activation of this signalling pathway. These observations lead us to speculate that activation of β-catenin signalling may artificially force cells toward commitment. Thus, the experimental paradigm used in this study may offer a means to examine the factors mediating differentiation, in particular, to the level of differentiation where SSC commitment becomes irreversible. Analyzing global gene expression profiles of the β-catenin signalling and non-signalling populations may provide information about the mechanism of SSC commitment at the molecular level.

How might Wnt3a stimulation support SSC maintenance? We found that manipulation with Wnt3a led to the formation of germ cell aggregations that resemble clusters, potentially as a result of activated cell cycle in non-SSCs. Interestingly, we note that tissue specific stem cells, which can proliferate ex vivo, have not been maintained alone but are always accompanied by differentiated daughter cells, such as seen in neurospheres and mammospheres [30], [31]. Neural stem cells are reported to represent ∼2% of total neurosphere cells [30], which suggests dependence by adult stem cells for cellular cues from non-stem progenitor cells to maintain stem cell functionality. This dependence is exemplified in ISC cultures, in which single stem cells produce crypt-like organoids composed of interacting ISCs and Paneth cells [16]. Paneth cells are specialized cells that are derived from stem cells and comprise the ISC niche by producing extrinsic factors to support ISCs. In vivo, a recent study pointed out a potential interplay between the stem cell pool and the progenitor pool to maintain the size of each cell population in balance [32].

Likewise, the formation of a germ cell cluster, which is an integrated community of interacting SSCs and daughter cells, may be an important in vitro phenomenon to support SSCs and may act as an in vitro SSC niche. It has been shown that constitutive activation of Ras-cyclin D2 in germ cells circumvents the need for GDNF and FGF2, yet induces cell division and cluster formation, leading to long-term SSC proliferation [33]. Even in this case, however, SSCs remained to be a minority population, implying that Ras-cyclin D2 also stimulated cell divisions that result in the production of mostly non-SSCs. Importantly, the frequency of SSCs after Ras-cyclin D2 stimulation did not differ from that reported for wild-type SSC clusters [33] which is relatively constant across studies reported thus far (1–3%) [5], [8], [9]. In our case, an SSC concentration was estimated to be 0.7–1.7% following Wnt3a stimulation (unpublished data). These results therefore argue that the SSC frequency is controlled to an optimal level in an in vitro germ cell cluster, perhaps through the communication among constituent cells of a cluster community. Thus, it is possible that added Wnt3a may induce cell divisions of committed progenitors and facilitate formation of clusters. Then this may indirectly result in the regulation of SSC numbers to an ideal level within a cluster population. On this basis, it is possible that SSCs may possess machinery that actively suppress β-catenin signalling, or these cells may not be equipped with the full range of cell-surface or intracellular mediators to transduce this signalling. Further studies are necessary to test this hypothesis of clusters acting as an in vitro SSC niche.

We previously reported that Wnt3a expression is not detected in the testis, implying that the in vitro effects observed in this study may be the result of functional redundancy with another Wnt. Functional redundancy has been shown by Wnt1, Wnt3a, or Wnt8 resulting in axis duplication during Xenopus gastrulation [34], . In the testis, we have previously detected the expression of classical β-catenin signalling class Wnts, notably Wnt1 and Wnt2b [7]. In vivo, Wnt1 expression is detected starting in the round spermatid stage however a role in spermatogenesis has not been well characterized [36]. Wnt2b also has no defined role in spermatogenesis however Wnt2b/β-catenin signalling has been implicated in kidney morphogenesis regulation in conjunction with Wnt11 and GDNF [37], [38]. Furthermore, Wnt2b has been shown to induce the proliferation of retinal progenitor cells in the avian eye [39]. In the testis, Wnt2b is detected at birth, is undetectable in adult, but is detected in cryptorchid testes suggesting that it may be expressed by spermatogonia.

In summary, this study demonstrates that Wnt3a activates the β-catenin pathway in committed progenitor cells and suggest that SSC maintenance can be promoted by the presence of aggregated progenitors. We observed that Wnt3a stimulated cell division of β-catenin signalling cells and stimulated community formation between SSCs and daughter cells, resulting in SSC maintenance. Therefore, these findings lead us to propose that differentiated daughter cells may be capable of contributing to the SSC regulatory function of the niche.

## Materials and Methods

### Donor Animals

Homozygous TCF/LEF-*lacZ* mice (from Dr. D. Dufort, McGill University) are on a CD-1 genetic background and carry the *lacZ* reporter gene driven by β-catenin-TCF/LEF responsive elements [21], [22]. B6ROSA mice are F_1_ hybrids of C57BL/6 (B6) and ROSA26 mice, which express the *lacZ* gene ubiquitously in virtually all cell types [23]. B6GFP mice (C57BL/6-Tg(CAG-EGFP)1Osb/J; The Jackson Laboratory) express GFP ubiquitously. Animal procedures were approved by the Animal Care and Use Committee of McGill University.

### Recipient Animals and Transplantation

Spermatogonial transplantation was performed and recipients were prepared as described previously [7]. Recipient mice for B6ROSA cells were 129/SvEv × B6 F_1_ hybrids, and those for TCF/LEF-*lacZ* cells, Ncr nu/nu mice (Taconic). Recipient animals were injected with busulfan i.p. (50 mg/kg for 129/SvEv × B6, 40 mg/kg for Ncr nu/nu) at 4–6 wk of age to eliminate endogenous spermatogenesis, at least 1 mo prior to transplantation. Donor cells were from cultures derived from 7–8 dpp prepubertal mouse testes. For transplantation, cultures were enzymatically digested to a single cell suspension and injected into the rete testis of recipient mice, to fill the seminiferous tubules. Recipient testes were analyzed for SSC quantification following staining with 5-bromo-4-chloro-3-indolyl β-d-galactoside (X-gal) 2 months post-transplantation. Colony numbers of donor-derived spermatogonesis were visually counted [24] and expressed as colonies/10^6^ cells initially placed in culture.

### Cell Culture

SSC cultures were established from immunomagnetic selected Thy1-positive testis cells from 7–8 days post partum (dpp) mice as described previously [8]. Cultures were maintained with a serum-free MEMα-based medium with supplements on a feeder layer of STO fibroblasts. Media were replenished every 3^rd^ day and clusters were subcultured onto freshly prepared STO feeder cells every 6–7 days. Cultures were maintained with “growth factors” consisting of GDNF (20 ng/ml), GFRa1 (75 ng/ml), and FGF2 (1 ng/ml) [4], [8]. Experiments were conducted using established cluster cells (>5 passages), removed from STO feeder cells using gentle pipetting, which results in an isolation of cluster cells at more than 90% purity [7]. Subsequently, clusters were digested to single cells following treatment with 0.05% trypsin-EDTA. For short-term feeder-free cultures, culture plates were coated with Matrigel (BD Biosciences), diluted 1∶2 in serum-free culture media, and incubated overnight at 4°C. The next day, excess Matrigel was removed and the plate was transferred to a 37°C humidified incubator, 30 min prior to use. For experiments, we used GDNF at 40 ng/ml, GFRα1 at 300 ng/ml, and FGF2 at 1 ng/ml, as in [4]. Recombinant Wnt3a and DKK1 (R&D Systems) were added at concentrations as previously reported [20]. All cultures were maintained at 37°C in a humidified incubator with 5% CO_2_.

To quantify TCF/LEF-*lacZ* expressing cells, clusters derived from reporter mice were stimulated with Wnt3a, reacted with X-gal overnight, and visually counted using a hemocytometer.

### Cluster-formation Analysis

As a short-term semi-quantitative assay for SSC activity, it has been determined that cluster number correlates with functional SSC number [8]. Hence, cluster cells exposed to experimental conditions were subcultured onto STO feeder cells in the presence of growth factors to induce cluster formation. Media were replenished 3 days after initial seeding and by day 6 cluster numbers were ready for quantification. Cluster numbers were determined in one of the following two ways. B6ROSA-derived clusters were reacted with X-gal overnight and cluster numbers were counted visually under a microscope. B6GFP-derived cluster numbers were acquired automatically using an ImageXpress^MICRO^ imaging system (Molecular Devices) as described previously [25]. Three experiments were performed for all cluster formation analyses and the average of at least two wells was recorded for each group per experiment. Cluster numbers were normalized to 10^6^ cells placed in culture.

### Flow Cytometric Analysis and Sorting

To isolate β-catenin-signalling cell populations following Wnt3a stimulation, TCF/LEF-*lacZ* clusters were reacted with 500 µM fluorescein di-β-D-galactopyranoside (FDG, Marker Gene Technologies) and sorted as described previously [7]. Experimental gates were established using control cells: B6ROSA (positive) and B6 (negative) cluster cells. For cell cycle analyses, B6ROSA cluster cells were fixed in 70% ethanol at −20°C overnight followed by incubation with 40 µg/ml propridium iodide (PI) and 100 µg/ml RNase at room temperature. Data were acquired on a FACScan or Accuri C6 Cytometer (Becton Dickinson), from three experiments with at least 10,000 events collected per sample.

### Cell Profiler Analysis

To quantify Wnt3a effects on germ cell clustering, B6GFP cluster cells were cultured feeder-free in a 96-well plate at 5×10^4^ cell/cm^2^ from 1 to 4 days in the presence or absence of Wnt3a. The time of cell seeding was deemed Day 0. Cultures were terminated each day from days 1 to 4 and, at each day, were reacted with a DNA dye (DAPI), to assist in the identification of cells. Thirteen representative fluorescent photomicrographs were taken from various points randomly in each culture well. For neighboring analyses we used the Cell Profiler Software Platform (Broad Institute) [26]. To this end, a pipeline was developed that identified individual cells based on their shape and expression of GFP and DAPI. Neighboring cells were defined as cells in physical contact and were identified as individual cells with adjacent cell boundary pixels to generate data on how many cells are associated with a given cell.

### Immunofluorescent Staining

B6ROSA cluster cells were cultured on Matrigel as above with Wnt3a (400 ng/ml). On day 4, cultures were fixed in 4% paraformaldehyde. Primary antibody used was mouse anti-LRP5/6 (R&D Systems). Secondary antibody used was goat anti-mouse conjugated-R-phycoerythrin (Jackson ImmunoResearch). DAPI was used to visualize cell nuclei.

### Quantitative PCR

Total RNA was extracted using the PicoPure RNA Isolation Kit (Applied Biosystems). Complementary DNA was synthesized using Superscript III Reverse Transcriptase (Invitrogen) with random hexamers. Quantitative PCR was performed with the QuantiTect SYBR Green PCR Kit (Qiagen) on a Rotogene 6000 (Corbett Research) with the program: 94°C for 15 min followed by 40 cycles of 94°C for 15 sec/51−60°C for 30 sec/72°C for 35 sec. Primer sequences were the following: c-Kit-F: TGGGAGTTTCCCAGAAACAG, c-Kit-R: AAATGGGCACTTGGTTTGAG; Nanos2-F: TGAGGTACCTGTCACCCACA, Nanos2-R: GGATCCTGAAGGCACAGAAA; Ngn3-F: CTCATTGGAGGAATTCCCTG, Ngn3-R: TTTCCACTAGCACCCACCAC; Plzf-F: GCAGCTATATTTGCAGTGAG, Plzf-R: TCTTGAGTGTGCTCTCATCC.

### Statistics

Data were expressed as mean ± SEM. Numbers of clusters in vitro and SSCs detected with spermatogonial transplantation were indicated as those per 10^6^ cells placed in culture, unless specified otherwise. Significance was determined using ANOVA followed by Fisher’s Test for Least Significant Difference. *p*<0.05 determined significance.

## Supporting Information

Figure S1 **Expression of spermatogonia markers in TCF/LEF-** ***lacZ*** **signalling populations.** Quantitative PCR on TCF/LEF-*lacZ*-negative cells (open bars), TCF/LEF-*lacZ* ^dim^ cells (shaded bars), and TCF/LEF-*lacZ* ^high^ cells (filled bars). TCF/LEF-*lacZ* ^high^ cells show lower expression of Plzf. Greater expression of the differentiating spermatogonia markers Ngn3 and c-kit are observed in the TCF/LEF-*lacZ* ^dim/high^ cell fractions. No significant difference was observed for any marker.
(EPS)

Figure S2 **CellProfiler demarcates individual cells in an aggregation.** Representative fluorescent photomicrographs of B6GFP cluster cells following control (top panels) or Wnt3a treatment (bottom panels) after 4 days. (**A–B)** Fluorescent images showing DAPI and GFP expression in cluster cells. Large and more densely growing “cluster-like” cell aggregations are found in Wnt3a-treated cultures compared to untreated controls. (**C**) Representative images from CellProfiler image analysis identifying individual cells. CellProfiler identifies cells based on the co-expression of DAPI and GFP and demarcates these individual cells using red outlines. (**D**) CellProfiler image analysis indicating numbers of neighboring cells. The color gradient labels individual cells according to the number of directly-contacted neighboring cells. Note the difference in the neighboring cell numbers indicated by the color gradient between control and Wnt3a groups. Scale bar: 48 µm.
(EPS)

## References

[1] de Rooij DG (2009). The spermatogonial stem cell niche.. Microsc Res Tech.

[2] Fuller MT, Spradling AC (2007). Male and female Drosophila germline stem cells: two versions of immortality.. Science.

[3] Brawley C, Matunis E (2004). Regeneration of male germline stem cells by spermatogonial dedifferentiation in vivo.. Science.

[4] Kubota H, Avarbock MR, Brinster RL (2004). Growth factors essential for self-renewal and expansion of mouse spermatogonial stem cells.. Proc Natl Acad Sci U S A.

[5] Oatley JM, Brinster RL (2008). Regulation of spermatogonial stem cell self-renewal in mammals.. Annu Rev Cell Dev Biol.

[6] Oatley JM, Oatley MJ, Avarbock MR, Tobias JW, Brinster RL (2009). Colony stimulating factor 1 is an extrinsic stimulator of mouse spermatogonial stem cell self-renewal.. Development.

[7] Yeh JR, Zhang X, Nagano MC (2011). Wnt5a is a cell-extrinsic factor that supports self-renewal of mouse spermatogonial stem cells.. J Cell Sci.

[8] Yeh JR, Zhang X, Nagano MC (2007). Establishment of a short-term in vitro assay for mouse spermatogonial stem cells.. Biol Reprod.

[9] Kanatsu-Shinohara M, Miki H, Inoue K, Ogonuki N, Toyokuni S (2005). Long-term culture of mouse male germline stem cells under serum-or feeder-free conditions.. Biol Reprod.

[10] Ebata KT, Yeh JR, Zhang X, Nagano MC (2011). Soluble growth factors stimulate spermatogonial stem cell divisions that maintain a stem cell pool and produce progenitors in vitro.. Exp Cell Res.

[11] Logan CY, Nusse R (2004). The Wnt signaling pathway in development and disease.. Annu Rev Cell Dev Biol.

[12] Reya T, Clevers H (2005). Wnt signalling in stem cells and cancer.. Nature.

[13] Haegebarth A, Clevers H (2009). Wnt signaling, lgr5, and stem cells in the intestine and skin.. Am J Pathol.

[14] Kalani MY, Cheshier SH, Cord BJ, Bababeygy SR, Vogel H (2008). Wnt-mediated self-renewal of neural stem/progenitor cells.. Proc Natl Acad Sci U S A.

[15] Reya T, Duncan AW, Ailles L, Domen J, Scherer DC (2003). A role for Wnt signalling in self-renewal of haematopoietic stem cells.. Nature.

[16] Sato T, van Es JH, Snippert HJ, Stange DE, Vries RG (2011). Paneth cells constitute the niche for Lgr5 stem cells in intestinal crypts.. Nature.

[17] Cobas M, Wilson A, Ernst B, Mancini SJ, MacDonald HR (2004). Beta-catenin is dispensable for hematopoiesis and lymphopoiesis.. J Exp Med.

[18] Scheller M, Huelsken J, Rosenbauer F, Taketo MM, Birchmeier W (2006). Hematopoietic stem cell and multilineage defects generated by constitutive beta-catenin activation.. Nat Immunol.

[19] Holowacz T, Huelsken J, Dufort D, van der Kooy D (2011). Neural stem cells are increased after loss of beta-catenin, but neural progenitors undergo cell death.. Eur J Neurosci.

[20] Golestaneh N, Beauchamp E, Fallen S, Kokkinaki M, Uren A (2009). Wnt signaling promotes proliferation and stemness regulation of spermatogonial stem/progenitor cells.. Reproduction.

[21] Mohamed OA, Clarke HJ, Dufort D (2004). Beta-catenin signaling marks the prospective site of primitive streak formation in the mouse embryo.. Dev Dyn.

[22] Mohamed OA, Jonnaert M, Labelle-Dumais C, Kuroda K, Clarke HJ (2005). Uterine Wnt/beta-catenin signaling is required for implantation.. Proc Natl Acad Sci U S A.

[23] Zambrowicz BP, Imamoto A, Fiering S, Herzenberg LA, Kerr WG (1997). Disruption of overlapping transcripts in the ROSA beta geo 26 gene trap strain leads to widespread expression of beta-galactosidase in mouse embryos and hematopoietic cells.. Proc Natl Acad Sci U S A.

[24] Zhang X, Ebata KT, Nagano MC (2003). Genetic analysis of the clonal origin of regenerating mouse spermatogenesis following transplantation.. Biol Reprod.

[25] Marcon L, Zhang X, Hales BF, Nagano MC, Robaire B (2010). Development of a short-term fluorescence-based assay to assess the toxicity of anticancer drugs on rat stem/progenitor spermatogonia in vitro.. Biol Reprod.

[26] Carpenter AE, Jones TR, Lamprecht MR, Clarke C, Kang IH (2006). CellProfiler: image analysis software for identifying and quantifying cell phenotypes.. Genome Biol.

[27] Nakagawa T, Nabeshima Y, Yoshida S (2007). Functional identification of the actual and potential stem cell compartments in mouse spermatogenesis.. Dev Cell.

[28] Nakagawa T, Sharma M, Nabeshima Y, Braun RE, Yoshida S (2010). Functional hierarchy and reversibility within the murine spermatogenic stem cell compartment.. Science.

[29] Morimoto H, Kanatsu-Shinohara M, Takashima S, Chuma S, Nakatsuji N (2009). Phenotypic plasticity of mouse spermatogonial stem cells.. PLoS One.

[30] Reynolds BA, Rietze RL (2005). Neural stem cells and neurospheres–re-evaluating the relationship.. Nat Methods.

[31] Dontu G, Abdallah WM, Foley JM, Jackson KW, Clarke MF (2003). In vitro propagation and transcriptional profiling of human mammary stem/progenitor cells.. Genes Dev.

[32] Aguirre A, Rubio ME, Gallo V (2010). Notch and EGFR pathway interaction regulates neural stem cell number and self-renewal.. Nature.

[33] Lee J, Kanatsu-Shinohara M, Morimoto H, Kazuki Y, Takashima S (2009). Genetic reconstruction of mouse spermatogonial stem cell self-renewal in vitro by Ras-cyclin D2 activation.. Cell Stem Cell.

[34] Kuhl M, Pandur P (2008). Dorsal axis duplication as a functional readout for Wnt activity.. Methods Mol Biol.

[35] Sokol S, Christian JL, Moon RT, Melton DA (1991). Injected Wnt RNA induces a complete body axis in Xenopus embryos.. Cell.

[36] Jen J, Deschepper CF, Shackleford GM, Lee CY, Lau YF (1990). Stage-specific expression of the lactate dehydrogenase-X gene in adult and developing mouse testes.. Mol Reprod Dev.

[37] Lin Y, Liu A, Zhang S, Ruusunen T, Kreidberg JA (2001). Induction of ureter branching as a response to Wnt-2b signaling during early kidney organogenesis.. Dev Dyn.

[38] Majumdar A, Vainio S, Kispert A, McMahon J, McMahon AP (2003). Wnt11 and Ret/Gdnf pathways cooperate in regulating ureteric branching during metanephric kidney development.. Development.

[39] Kubo F, Takeichi M, Nakagawa S (2005). Wnt2b inhibits differentiation of retinal progenitor cells in the absence of Notch activity by downregulating the expression of proneural genes.. Development.

